# Nanotechnology-Based Strategies for Safe and Effective Immunotherapy

**DOI:** 10.3390/molecules29245855

**Published:** 2024-12-11

**Authors:** Seeun Hong, Juwon Park, Yoojeong Oh, Hanhee Cho, Kwangmeyung Kim

**Affiliations:** Graduate School of Pharmaceutical Sciences, College of Pharmacy, Ewha Womans University, Seoul 03760, Republic of Korea; hongseeun@ewhain.net (S.H.); parkjoowon@ewhain.net (J.P.); ubellro@ewhain.net (Y.O.); ricky@ewha.ac.kr (H.C.)

**Keywords:** drug delivery, cancer treatment, nanoparticle, immunotherapy, chemotherapy, photodynamic therapy, photothermal therapy, radiotherapy

## Abstract

Cancer immunotherapy using immune checkpoint blockades has emerged as a promising therapeutic approach. However, immunotherapy faces challenges such as low response rates in solid tumors, necessitating strategies to remodel the immune-suppressive tumor microenvironment (TME) into an immune-activated state. One of the primary approaches to achieve this transformation is through the induction of immunogenic cell death (ICD). Herein, we discussed strategies to maximize ICD induction using nanoparticles. In particular, this review highlighted various studies integrating chemotherapy, radiation therapy (RT), photodynamic therapy (PDT), and photothermal therapy (PTT) with nanoparticle-based immunotherapy. The research covered in this review aims to provide valuable insights for future studies on nanoparticle-assisted immunotherapy.

## 1. Introduction

Cancer immunotherapy has gained significant attention as a revolutionary strategy in oncology for achieving the complete cure of cancer and has been broadly applied regardless of cancer type or stage [1,2,3]. The major difference between immunotherapy and conventional cancer treatments lies in the fact that immunotherapy activates the patient’s own immune system to treat even cancers that cannot be treated through surgery or chemotherapy [4,5]. While traditional chemotherapy is limited by non-specific toxicity and the challenge of treating metastatic cancer due to the lack of selectivity and acquired resistance, immunotherapy can target not only the primary tumor but also metastatic sites through the activation of the adaptive immune response [6,7,8,9]. These treatments typically inhibit the function of immune checkpoint proteins such as programmed cell death protein 1 (PD-1), programmed cell death ligand 1 (PD-L1), and cytotoxic T-lymphocyte-associated protein 4 (CTLA-4), which play a key role in immune evasion [10,11,12]. By blocking, degrading, or suppressing the generation of these immune checkpoint proteins, immunotherapy allows cytotoxic T cells to recognize and eliminate tumor cells more effectively [13,14].

Despite this remarkable performance, immunotherapy still confronts many hurdles such as low patient response rates, high costs, and toxicity related to self-immunity [15,16]. Among these, the low response rate is a primary challenge that must be addressed, as it is a common issue in the majority of solid tumors [17,18]. Unlike hematological tumors, immune responses in solid tumors are mainly driven by antigen presentation through dendritic cells and macrophages [19,20,21]. However, the immunosuppressive TME induces the upregulation of regulatory T cells (Tregs), myeloid-derived suppressor cells (MDSCs), vascular endothelial growth factor (VEGF), and transforming growth factor β (TGF-β), leading to the M2 polarization of macrophages, which further promotes an immunosuppressive state [22,23]. Therefore, remodeling the TME to create an immune-activated environment could serve as an effective approach to overcoming the low response rate [24,25].

Inducing ICD in tumor cells is one approach to remodeling the TME [24,26]. Well-known ICD inducers include anthracycline-based drugs like doxorubicin (DOX), as well as treatments such as PDT, RT, and PTT [27]. These treatments generate stress, such as DNA damage and reactive oxygen species (ROS) production, leading to ICD, which induces damage-associated molecular pattern (DAMP) signals [28,29]. DAMPs include immunogenic signals, such as high-mobility group box 1 (HMGB1), calreticulin (CRT), and adenosine triphosphate (ATP) [30]. Each signal plays a distinct role in activating immune responses. For instance, HMGB1 suppresses immunosuppressive components such as Tregs and MDSCs through the toll-like receptor 4 (TLR4) pathway, while enhancing immune-inflammatory responses within the tissue [31]. CRT, acting as an “eat-me” signal, is translocated to the cell surface via the eukaryotic initiation factor 2 α-activating transcription factor 4-C/EBP homologous protein (eIF2α-ATF4-CHOP) pathway, thereby promoting the polarization of macrophages toward the M1 phenotype [32,33]. ATP functions as a “find-me” signal, recruiting immune cells to sites of inflammation [34]. Through this cascade of events, dendritic cells are activated and matured, enabling the presentation of tumor antigens via both MHC class I and II pathways [35]. As a result, this can effectively resolve issues like low T lymphocyte infiltration caused by immunosuppressive conditions in tumors, enhancing the efficacy of immune checkpoint inhibitors (ICIs) in solid tumors [36,37].

However, the non-specific toxicity due to the low specificity and targeting efficiency of ICD inducers are significant challenges for combination treatment with ICIs [38,39]. The low specificity and lack of targeting efficiency prevent sufficient delivery of ICD inducers to tumor sites, hindering the effective induction of the ICD required for immune activation [40]. Furthermore, attempting to resolve this issue with high doses may lead to severe systemic side effects and non-specific toxicity to immune cells [39]. This could result in inadequate immune responses, even if ICD is induced, due to the depletion or dysfunction of immune cells. Thus, optimizing the dosage is crucial; therefore, the drug delivery system using nanoparticles such as peptides, proteins, polymeric, inorganic, dendrimers, or liposomes presents a promising approach to overcoming these challenges [41,42,43,44,45,46,47,48]. First, nanoparticles can enhance drug efficacy by taking advantage of the enhanced permeation and retention (EPR) effect, which leads to tumor accumulation due to the leaky blood vessels caused by tumor angiogenesis [49,50]. In addition to delivering drugs, the surface of nanoparticles can be easily modified with molecules, peptides, or proteins to target tumor-specific receptors, thereby improving targeting efficiency and providing additional synergistic effects with encapsulated drugs [51,52]. This strategy enables specific targeting of tumor tissue, which minimizes the non-specific toxicity of ICD inducers and immunotherapeutic agents resulting in the maximization of drug efficacy and, as a result, the reduction of the relative cost [53,54]. Moreover, it facilitates effective TME remodeling, helping to overcome immunosuppressive conditions and improving the overall immune response, which can help address the low response rates of currently used ICIs in clinical settings [55,56].

**Scheme 1:**
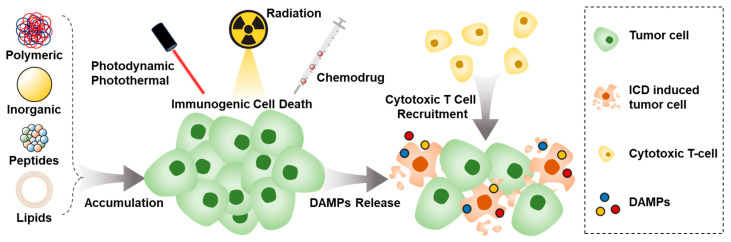
Schematic illustration of ICD-inducing mechanisms through chemotherapy, PDT, RT, and PTT utilizing nanoparticles. Several nanoparticles allow for the accumulation of encapsulated drugs in the tumor tissue, and induced ICD remodeled immune-suppressive TME to an immune-activated environment through the DAMP release resulting in enhanced T cell recruitment to the tumor tissue.

Herein, we propose the study of nanoparticles for remodeling the TME by introducing ICD-inducing treatments, such as chemotherapy, PDT, RT, and PTT (Scheme 1). These studies successfully remodeled the TME via inducing ICD, transforming the immunosuppressive condition into an immune-activated environment [57]. In addition to addressing methods for overcoming immunosuppressive conditions, we present future perspectives on nanoparticle-based immunotherapy, providing deep insights for future research into TME remodeling using nanoparticles.

## 2. Chemotherapy with Nanoparticles for ICD

Chemotherapy is one of the most effective approaches for ICD [58,59]. Several chemotherapeutic agents, such as doxorubicin, mitoxantrone, and oxaliplatin, are known to induce ICD [60]. For instance, doxorubicin induces cell death by causing DNA damage and generating ROS, ultimately leading to apoptosis [61]. This process triggers sufficient endoplasmic reticulum (ER) stress through an intrinsic pathway and extrinsic pathway, finally activating the protein kinase RNA-like endoplasmic reticulum kinase (PERK)-eukaryotic initiation factor 2α (elF2α)-C/EBP homologous protein (CHOP) pathway for promoting cell apoptosis [62,63,64]. These processes effectively induce ICD; therefore, combining doxorubicin with immunotherapy can enhance the anti-tumor immune response and improve treatment efficacy [65,66,67].

**Figure 1:**
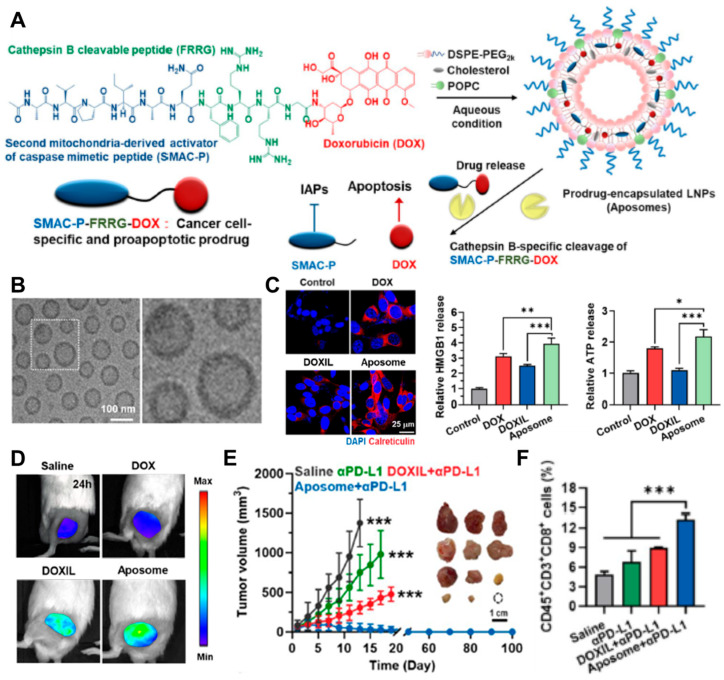
(**A**) Schematic illustration of the cancer-specific and proapoptotic prodrug SMAC-P-FRRG-DOX loaded into LNPs. (**B**) TEM images of Aposomes with a size of 100 nm. (**C**) The release of DAMPs (CRT, HMGB1, ATP) after DOX, DOXIL, or Aposome treatment. (**D**) In vivo fluorescence images after DOX, DOXIL, and Aposome treatment in the CT26 tumor xenograft model. (**E**) The tumor growth curve of the CT26 mouse model treated with saline, a PD-L1 antibody, DOXIL with a PD-L1 antibody, and Aposome with a PD-L1 antibody. (**F**) The relative CD8^+^ T cell expression level in excised tumor tissue on day 13. Significance was determined by the Tukey−Kramer post hoc test (* *p* < 0.05, ** *p* < 0.01, *** *p* < 0.001). Reproduced with permission [68]. Copyright: BMC, 2024.

Kim et al. developed a liposome-encapsulated prodrug composed of an inhibitor of apoptosis protein (IAP)-inhibiting peptides and DOX to enhance ICD through synergistic effect for maximizing the therapeutic effect of combination therapy with PD-L1 antibody (Figure 1A) [68]. They conjugated peptide sequences, including a second mitochondria-derived activator of caspase (SMAC) mimetic peptide (AVPIAQ) and cathepsin B cleavable peptide (FRRG), to doxorubicin, and then encapsulated in PEGylated liposomes (Aposome). The formulated liposomes were analyzed through dynamic light scattering (DLS) and transmission electron microscope (TEM) with an average size of 100 nm (Figure 1B). Aposome was only activated in tumor cells due to the cancer-overexpressed cathepsin B with no toxicity observed in normal cells and immune cells. Owing to the synergistic effects of SMAC and DOX, higher expression levels of DAMPs such as CRT, HMGB1, and ATP in Aposome-treated CT26 tumor cells were observed compared to DOX- and DOXIL-treated groups (Figure 1C). They also demonstrated the enhanced targeting efficiency of Aposome compared to DOX, due to the EPR effect induced by liposome formulation (Figure 1D). As a result, the Aposome group treated with a PD-L1 antibody showed improved tumor growth suppression with an 80% clearance rate (Figure 1E), and the upregulation of cytotoxic CD8^+^ T cells was observed from excreted tumor tissues in Aposome groups treated with PD-L1 antibody (Figure 1F).

Yang and colleagues investigated paclitaxel (PTX)-induced ICD to improve the immune response of the PD-1 antibody [69]. They introduced methoxy-poly(ethylene glycol)-*b*-poly(D, L-lactide) (mPEG-PDLLA) to formulate PTX-encapsulated nano-micelle (Nano-PTX) for enhancing tumor-targeting efficiency and reducing non-specific toxicity. As expected, the Nano-PTX-treated group showed enhanced CD8^+^ cytotoxic T cells in excised tumor tissue from CT26-bearing mice compared to the control group. They demonstrated the release of DAMPs in Nano-PTX-treated CT26 colon cancer cells such as CRT and HMGB1 as the reason for the enhancement of CD8^+^ T cells, which results in the upregulation of phagocytosis and immune-active cytokines. Based on these results, the PD-1 antibody was co-administered with Nano-PTX in the MC38 mouse model, resulting in a 78% tumor clearance rate compared to the control group and monotherapy groups. Furthermore, when a rechallenge was performed on the models with tumor clearance, none of the mice exhibited tumor growth, which was presumed to be due to immune memory.

Zhang et al. developed DOX and PD-L1 blockade peptide P-12 (FPNWSLRPMNQM) encapsulated lipids/poly(lactic-co-glycolic acid)(PLGA) dual composition nanoparticles for ICD-induced immunotherapy [70]. They established the nanoparticle composition with optimal drug encapsulation and release profiles with a size of 100–125 nm, and finally optimized lipid/PLGA nanoparticles with 30% of DSPE-PEG_5000_ and 10% of cell penetration peptide R8-conjugated DSPE-PEG_2000_. DOX and P-12 were encapsulated in optimized lipid/PLGA nanoparticles (LPN-30-R8^2k^) and were efficiently absorbed into CT26 cells, inducing ICD and resulting in the upregulation of CRT as a DAMP. The LPN-30-R8^2k^ effectively targeted the tumor tissue in a CT26-bearing mouse model via IV injection, due to the EPR effect of the nanoparticle formulation. As expected, enhanced tumor growth suppression was observed in the LPN-30-R8^2k^-treated CT26 mouse model, and the upregulation of cytotoxic T lymphocytes and the downregulation of Tregs were analyzed from excised tumor tissue compared to other groups due to enhanced DAMPs being released by encapsulated DOX.

Song and colleagues developed surface-modified chitosan nanoparticles (CNP) encapsulating chemotherapeutic agents [71]. They prepared CNP by introducing a PD-L1 peptide antagonist onto their surface (PP-CNP), which blocked the cancer immune checkpoint protein PD-L1 via a multivalent binding mechanism. Additionally, the CNPs encapsulated DOX as an ICD inducer and were delivered to the tumor site via the EPR effect. The synergistic effect of DOX and PD-L1 peptide blockade led to tumor clearance 60 days after the first subcutaneous inoculation of CT26 cancer cells in mouse flank accompanied by an activated immune response, including enhanced CD8^+^ T cell and downregulated CD25^+^ Tregs. Furthermore, DOX-loaded PP-CNP showed improved therapeutic efficacy in a lung metastasis model, enabling survival of the treated mice at the end of the treatment, whereas all of the other groups exhibited a zero-survival rate.

## 3. PDT with Nanoparticles for ICD

PDT is a treatment method that uses light-sensitive compounds called photosensitizers (PS) such as verteporfin, chlorin e6, zinc phthalocyanine, and aluminum phthalocyanine tetrasulfonate, which cause effective ICD for cancer immunotherapy [72,73]. When PS absorbs specific wavelengths of light, the energy level within the PS rises from the ground state to the excited state [74,75]. During the process of returning to the ground state, the PS interacts with surrounding molecules such as oxygen to generate various ROS through both type I and type II mechanisms [75,76,77]. Similarly to the mechanism of chemotherapy, the generated ROS induces cell death by triggering ER stress, leading to significant ICD [78,79]. As a result, DAMPs are secreted from apoptotic cells, allowing the immune system to recognize and attack tumor cells efficiently [80,81,82].

**Figure 2:**
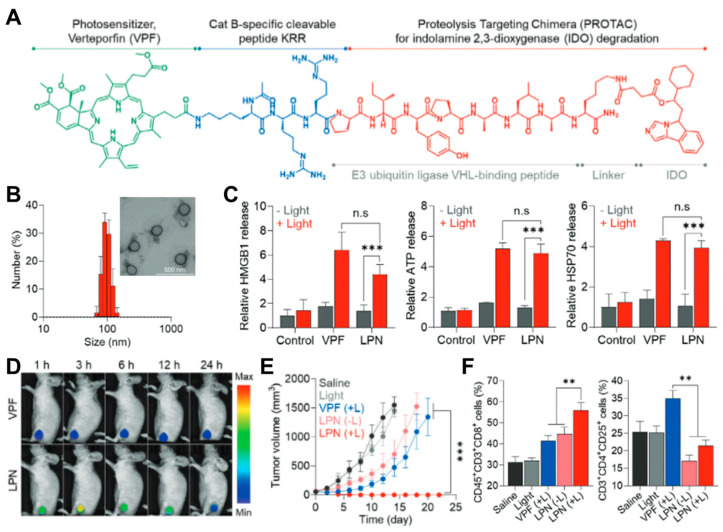
(**A**) Chemical structure of cathepsin-B activatable VPF-IDO PROTAC prodrug. (**B**) TEM images of LPNs with size of 102.49 ± 7.39 nm confirmed by DLS. (**C**) Relative DAMP expression level after treatment of VPF or LPN, with or without light irradiation. (**D**) Fluorescence images of CT26-bearing mouse model treated with VPF or LPN. (**E**) Tumor growth curve after treatment of light irradiation, VPF with light irradiation, and LPN with or without light irradiation. (**F**) Relative expression level of CD8^+^ and CD25^+^ cells in excised tumor tissue. Significance was determined by one-way ANOVA with Tukey–Kramer post hoc test (** *p* < 0.01, *** *p* < 0.001). Reproduced with permission [83]. Copyright: Wiley-VCH GmbH, 2024.

Choi et al. conjugated photosensitizer verteporfin (VPF) and indolamine 2,3-dioxygenase (IDO) degradable proteolysis targeting chimera (PROTAC) through tumor-overexpressed enzyme cathepsin B cleavable linker peptide (KRR) to form light-triggered PROTAC nanoassemblies (LPNs) (Figure 2A) [83]. The LPNs were 102.49 ± 7.39 nm in size with a spherical structure (Figure 2B); they maintained their size for 48 h. Due to the ROS generation via light irradiation to verteporfin in the LPNs, DAMP secretions such as HMGB1, ATP, and HSP70 were upregulated in CT26 tumor cells compared to the treatment without light (Figure 2C). Moreover, due to the spherical structure with an appropriate size for inducing the EPR effect, LNPs showed enhanced tumor-targeting efficiency in the CT26 tumor-bearing mouse model relative to the verteporfin-treated group (Figure 2D). The LPNs with light irradiation showed a 100% clearance rate (n = 5) (Figure 2E) resulting from improved CD8^+^ T cell activity and impeded CD25^+^ Tregs activity due to the induced ICD (Figure 2F). In addition, there was no tumor recurrence when CT26 tumor cells were inoculated into tumor clearance mouse species, and it is due to the adaptive immunity by CD44+/CD62L^low^ memory T cell.

Wang et al. designed a PDT-immunotherapy system by utilizing membrane-coated nanoparticles (MON) to amplify antitumor immune responses through ROS generation [84]. They focused on spatially packaging antigen ovalbumin (OVA) for encapsulating photosensitizer Ce6 to construct an immunogenic and ICD-inducible nanoparticle. These nanoparticles were formulated by loading the photosensitizer Ce6 to OVA complex, coated with the membrane of B16-OVA cells. The nanoparticles showed a final size of approximately 85 nm, enabling effective tumor targeting. Upon laser irradiation, the ROS generated by Ce6-induced ICD released DAMPs such as CRT and HMGB1, which enhanced phagocytosis and cytokine production. In vivo experiments using B16-OVA tumor models demonstrated that MON treatment achieved complete tumor regression and prevented metastasis through an abscopal effect. Additionally, MON therapy promoted a strong immune response, with increased CD8^+^ T cells and reduced Tregs, establishing immune memory that effectively prevented tumor recurrence upon rechallenging.

Zhao and colleagues investigated PDT combined with PTT using indocyanine green (ICG)-loaded nanoaggregates (ICG-DNA) to induce ICD and enhance systemic antitumor immunity [85]. The nanoparticles were formulated by coordinating ICG with a thermosensitive polymer, *p*(MEO_2_MA_160_-*co*-OEGMA_40_)-*b*-*p*SS_30_ (POEGS), resulting in an average particle size of 197 nm (TEM) and 246.8 nm (DLS). While 808 nm light irradiation, ICG-DNA produced singlet oxygen (^1^O_2_), triggering the release of DAMPs such as CRT and HMGB1, resulting in activated phagocytosis and promoting immune-active cytokine expression. In a bilateral 4T1 tumor model, treatment with ICG-DNA (P/I = 6.55) effectively eradicated primary tumors and exhibited an abscopal effect by inhibiting distant tumor growth. The enhanced immune response was evidenced by increased CD8^+^ cytotoxic T cells and reduced Tregs, along with higher levels of mature dendritic cells (DCs), leading to a robust antitumor immune response and preventing tumor recurrence during the rechallenge test.

Jin et al. tried to address the limitations of traditional PDT, such as the low tissue penetration of visible light required for ROS generation and poor bioavailability, by utilizing lanthanide-doped upconversion nanoparticles (UCNPs) [86]. Additionally, they employed a click reaction to conjugate rose bengal (RB) and ROS-specific release-capable DOX onto UCNPs. The nanoparticles were then coated with cell membranes extracted from 4T1 cancer cells, providing immune evasion properties and enhancing tumor accumulation. Upon applying 980 nm NIR irradiation, which can penetrate deep tissues, the UCNPs emitted visible light luminescence, triggering ROS generation by the encapsulated RB. This effectively induced cell death within the tissue. Moreover, the ROS-triggered release of DOX caused a synergistic ICD induction. In a lung metastasis animal model, the combination treatment with an antibody against CD73, which inhibits T cell infiltration, further amplified immune responses, including the upregulation of CD8^+^ T cells and CD86^+^ cells and the downregulation of Tregs. This approach resulted in a 93.4% reduction in tumor growth compared to the control group.

## 4. RT with Nanoparticles for ICD

RT is another strategy to induce ICD, transforming immunosuppressive TME into an immune-activated condition [87]. RT is a cancer treatment method that uses ionizing radiation to damage the DNA of tumor cells, leading to their destruction [88,89]. The ionizing radiation generates ROS and causes direct DNA double-strand breaks, resulting in severe cellular damage and apoptosis [90,91]. DNA damage and ROS-induced ER stress trigger cell death mechanisms similar to those previously mentioned, making RT capable of effectively inducing ICD [92,93,94]. Therefore, the combination of localized radiotherapy and immunotherapy could be utilized as an efficient immunotherapeutic strategy while minimizing side effects for patients [95,96,97].

**Figure 3:**
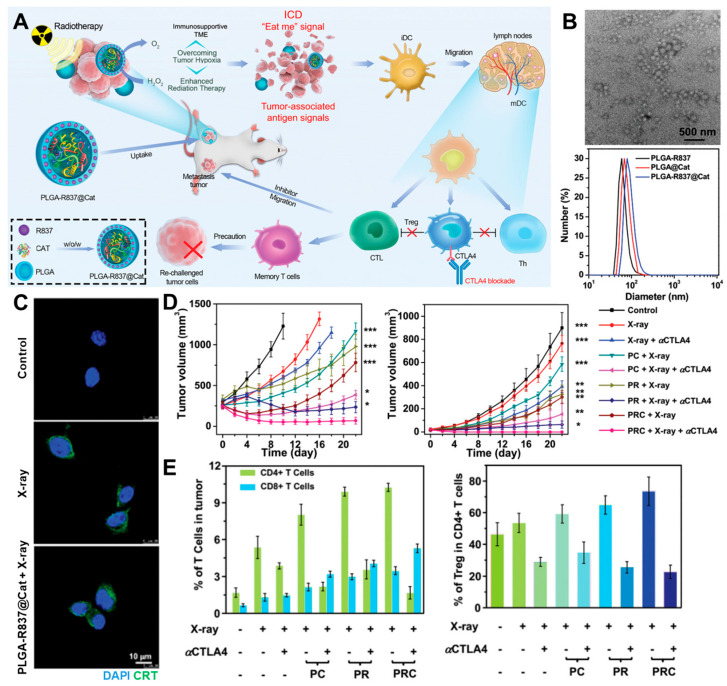
(**A**) Schematic illustration of PLGA-R837@Cat. (**B**) TEM images of PLGA-R837@Cat and DLS confirmation with a size of 100 nm. (**C**) Fluorescence images of CRT exposure after X-ray irradiation or PLGA-R837@Cat treatment with X-ray irradiation. (**D**) The growth curve of a primary tumor and a secondary inoculated tumor with various treatment conditions. (**E**) The relative expression level of T cells and Tregs at the excised tumor tissue. Significance was determined by one-way ANOVA using the Tukey post-test. (* *p* < 0.05, ** *p* < 0.01, *** *p* < 0.001). Reproduced with permission [98]. Copyright: Wiley-VCH GmbH, 2019.

For robust cancer immunotherapy, Chen et al. combined radiotherapy as a strong ICD-inducing strategy [98]. They developed PLGA nanoparticles which encapsulated water-soluble catalase (Cat) as an oxygen supplier and imiquimod (R837) as a toll-like receptor 7 agonist and triggered potent ICD through radiation exposure for enhanced treatment efficiency of CTLA-4 antibody (Figure 3A). The Cat and R837 containing PLGA nanoparticles (PLGA-R837@Cat) were observed via TEM and DLS, showing a homogeneous size of around 100 nm as spherical structures (Figure 3B). In addition, PLGA-R837@Cat-treated tumor tissue escaped from hypoxia condition compared to control and PLGA-R837-treated groups overcoming insufficient oxygen supply, which is one of the current challenges of radiotherapy. Also, due to the CRT expression found by X-ray irradiation (Figure 3C), primary tumors and secondary inoculated tumors with the PLGA-R837@Cat treatment combined with CTLA-4 antibody showed complete tumor regression (Figure 3D). As expected, improved CD8^+^ cytotoxic T cells and suppressed Tregs in CD4^+^ T cells were observed in PLGA-R837@Cat with CTLA-4 antibody-treated tumor tissues compared to other groups (Figure 3E).

Wang et al. developed oxaliplatin/Fe (OXA/Fe) bimetallic nanoparticles as cascade-sensitizing amplifiers to enhance low-dose radio-immunotherapy [99]. The size of nanoparticles was shown 106.6 nm with a spherical morphology, which was confirmed by DLS and TEM. These nanoparticles exhibited increased X-ray sensitivity through encapsulated Fe; thus, ROS generation was amplified, inducing strong ICD with enhanced CRT exposure and HMGB1 release as evidence. Intravenous injection of OXA/Fe NPs in a CT26 tumor xenograft model followed by low-dose X-ray irradiation resulted in the effective regression of both primary and abscopal tumors, with heightened CRT exposure in excised primary tumor and superior CD8^+^ T cell activity in the abscopal tumors. This treatment not only promoted tumor regression but also established immune memory, preventing metastasis and recurrence.

The nanoscale coordination polymers composed of gadolinium, 5′-Guanosine monophosphate, and Hemin (H@Gd-NCPs) were developed by Huang et al. [100]. The efficacy of radiation was enhanced in two ways. First, the encapsulated gadolinium amplified X-ray absorption leading to effective immunogenic cell death. Second, Hemin exhibited peroxidase-mimetic activity, providing an H_2_O_2_ supply to overcome the hypoxic tumor microenvironment. As a result, the H@Gd-NCPs with 112.5 nm size showed enhanced tumor growth suppression in the CT26 xenograft model compared to other groups when combined with X-ray radiation. In addition, CRT and HMGB1 secretion were detected from excised tumor tissue, with the highest expression levels detected in the H@Gd-NCPs treated group following radiation. H@Gd-NCPs with radiation and an anti-PD-L1 antibody was further treated in the abscopal model, resulting in a significant increase in CD8⁺ T cells and interferon-γ (INF-γ) at both the primary and distant tumors, leading to 37.5% tumor clearance at the distant site.

Guan et al. developed a PEGylated hollow manganese dioxide (HMP)-based nanoplatform for enhanced post-surgical RT and immunotherapy [101]. The system combines the hypoxia-relieving properties of MnO_2_, which decomposes tumor-derived H_2_O_2_ into oxygen, with the tumor-targeting delivery of a PI3Kγ inhibitor (IPI549) to reduce MDSCs and reprogram the TME. This approach improves RT efficacy by enhancing immunogenic cell death and sensitizes tumors to anti-PD-L1 therapy. In murine models of colon and melanoma cancer, the combination therapy significantly inhibited residual and metastatic tumor growth, induced strong immune memory, and achieved complete protection against tumor rechallenging in 100% of treated mice. The strategy demonstrates the potential of nanosystems for synergistic radioimmunotherapy and immune modulation.

## 5. PTT with Nanoparticles for ICD

PTT is another effective treatment for inducing ICD [102]. PTT utilizes nanoparticles or other heat-sensitive agents such as gold nanoparticles, graphene oxide, and carbon nanotubes, and these agents generate localized heat upon exposure to near-infrared light [103,104,105,106]. Electron clouds in these materials are excited by absorbed light, and this phenomenon is known as plasmon resonance [107]. After excitation, the energy level of the electron cloud decreases to the ground state, with energy transfer inducing moderate heating in the range of 41 to 47 °C [108,109,110]. The heat from energy transfer causes selective tumor eradication due to its poor blood supply compared to normal tissue [111]. This heat-induced cell death also triggers the release of DAMPs, leading to immune cell activation through induced ICD [112,113]. As a result, combining PTT with immunotherapy can amplify the anti-tumor immune response and improve the overall treatment outcomes [102,114,115].

**Figure 4:**
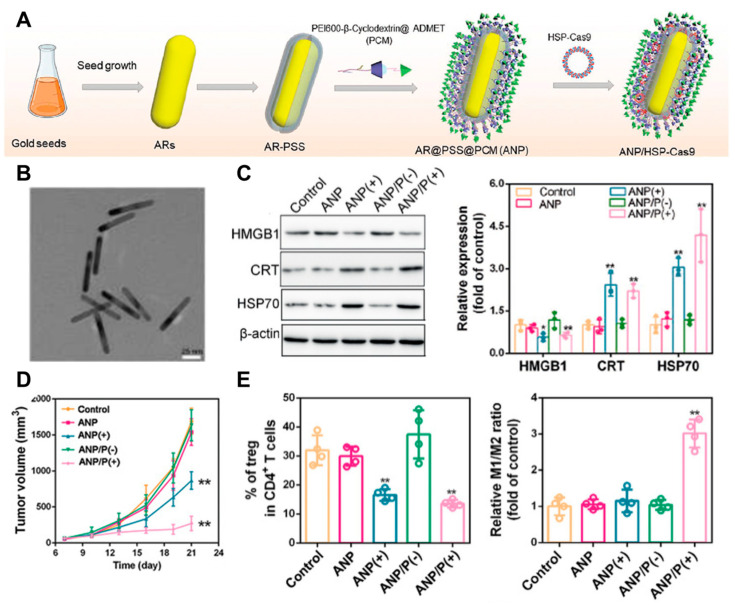
(**A**) Schematic illustration of ANP/HSP-Cas9 preparation. (**B**) TEM images of AR. (**C**) Western blot images of DAMPs (HMGB1, CRT, HSP70) after treatment and its quantification. (**D**) Tumor growth curve with the treatment of ANP, ANP with light, and ANP/P with or without light. (**E**) The relative expression level of Tregs and M1/M2 ratio at the tumor tissue. The significance was determined by Student’s *t*-test (**C**,**E**) or one-way ANOVA using the Tukey post-test (**D**). (* *p* < 0.05, ** *p* < 0.01). Reproduced with permission. Copyright: Wiley-VCH GmbH, 2021.

Tang et al. developed gold nanorods (AR) and CRISPR/Cas9 to induce ICD through the photothermal effect and disrupt PD-L1 for achieving effective immunotherapy [116]. They fabricated double-coated cationic gold nanorods (ANP) using polystyrene sulfonate (PSS) and polyethyleneimine-modified β-cyclodextrin with biguanidyl adamantane (PCM), enabling the intracellular delivery of a CRISPR/Cas9 plasmid with a heat-inducible promoter (HSP-Cas9) (Figure 4A). The HSP-Cas9 activated the transcription of Cas9 and its associated sgRNA upon exposure to mild hyperthermia, then silencing PD-L1 at a genomic level. The structure of the synthesized AR was observed by TEM, revealing a rod structure with a length of 60.14 nm and a width of 8.02 nm (Figure 4B). Moreover, the photothermal effect, generated by the gold nanostructure upon light irradiation-induced ICD, is evidenced by the release of HMGB1 and the exposure of CRT and HSP70 in B16F10 cancer cells (Figure 4C). As expected, the group treated with ANP/HSP-Cas9 (ANP/P) and light irradiation exhibited the most effective tumor regression, attributed to both ICD and PD-L1 disruption caused by the plasmid (Figure 4D). This result was confirmed by a reduced Treg ratio and an increased M1/M2 ratio in the excised tumor (Figure 4E).

Meanwhile, Li et al. conducted a study on a dual immunotherapy model combining PTT and PDT [79]. They developed ER-targeting pardaxin (FAL) peptides and indocyanine green (ICG) conjugated hollow gold nanospheres (FAL-ICG-HAuNS). Additionally, they designed hemoglobin liposomes conjugated with FAL (FAL-Hb-lipo) to overcome hypoxic conditions. The sizes of FAL-ICG-HAuNS and FAL-Hb-lipo were 151 ± 4.6 nm and 160.5 ± 6.6 nm, respectively. Due to rapid cellular internalization and high stability facilitated by FAL, both particles exhibited strong tumor accumulation and retention. The combination therapy of FAL-ICG-HAuNS and FAL-Hb-lipo maximized tumor growth suppression following laser irradiation, attributed to the high tumor-targeting ability, PDT effect of ICG, PTT effect of HAuNS, and oxygen delivery by Hb. These outcomes were further validated by the exposure of CRT in the tumor, which led to immune cell activation such as CD8^+^ T cells and the upregulation of immune cytokines such as INF- γ and IL-6.

Chen et al. developed nanoparticles by templating manganese dioxide (MnO_2_) with bovine serum albumin (BSA) and conjugating it with the anticancer drug DOX (DOX-BSA/MnO_2_) [117]. The developed DOX-BSA/MnO_2_ nanoparticles formed spherical structures with a size of 32.5 ± 2.31 nm, which enabled higher tumor-targeting efficiency compared to free drugs. Additionally, when DOX-BSA/MnO_2_ combined with laser irradiation was applied to 4T1 cells, the results demonstrated effective ICD induction, as evidenced by increased CRT exposure and the release of ATP and HMGB-1 compared to other groups. Based on these results, combination therapy with DOX-BSA/MnO_2_ and laser irradiation in a 4T1 tumor mouse model resulted in significantly enhanced therapeutic efficacy compared to other treatment groups. This outcome was further confirmed by increased M1 polarization, enhanced CD8⁺ T cell infiltration, and decreased Treg levels in tumors extracted from the DOX-BSA/MnO_2_ with the laser treatment group. The therapeutic potential was further validated through an abscopal model, demonstrating the effectiveness of the treatment.

Yun et al. developed silica-coated gold nanorods (AuNR@SiO_2_) designed for mild-condition photothermal therapy (PTT) [118]. They identified the difficulty in achieving precise temperature control optimized for immunotherapy as a key limitation of traditional PTT. To address this issue, they optimized the temperature by varying the thickness of the silica coating and treatment dosage of AuNR@SiO_2_. Specifically, silica nanorods with a thickness of 20 nm (AuNR@SiO_2_-20) demonstrated precise temperature control. When administered at different doses, 100 μg/kg of AuNR@SiO_2_ induced a high proportion of early apoptosis, a condition favorable for active ICD. As a result, there was an enhanced release of DAMPs compared to the control group. When combined with PD-L1 antibodies, this approach achieved a 100% tumor clearance rate, demonstrating exceptional therapeutic efficacy. Furthermore, when cancer cells were re-inoculated into the cleared CT26 mouse models, tumor regression was observed in naive mice but not in the treated mouse models, indicating the acquisition of immune memory properties due to immune activation.

## 6. Conclusions

As discussed earlier, the use of nanoparticles enables effective TME remodeling through the induction of ICD, facilitating complete tumor regression. The induction of ICD becomes more efficient and tumor-targeted by integrating nanoparticles with various treatments such as chemotherapy, PDT, radiation, and PTT. Furthermore, ongoing research aims to combine multiple therapeutic approaches, such as chemo-photodynamic therapy and chemo-radiation therapy, within a single nanoparticle platform. In the future, these advances are expected to lead to the development of a total solution, where a single nanoparticle carries customized therapeutic agents and methods tailored to individual patients. With continuous innovation and research, these technologies are anticipated to maximize the efficacy of PD-L1 antibodies in solid tumors and open new horizons in cancer treatment.

## Data Availability

No new data were created or analyzed in this study.
